# Determination of Thermal Properties of Mineral Wool Required for the Safety Analysis of Sandwich Panels Subjected to Fire Loads

**DOI:** 10.3390/ma16175852

**Published:** 2023-08-26

**Authors:** El Mehdi Ablaoui, Michał Malendowski, Wojciech Szymkuc, Zbigniew Pozorski

**Affiliations:** Faculty of Civil and Transport Engineering, Poznan University of Technology, 60-965 Poznan, Poland; elmehdi.ablaoui@doctorate.put.poznan.pl (E.M.A.); michal.malendowski@put.poznan.pl (M.M.); wojciech.szymkuc@put.poznan.pl (W.S.)

**Keywords:** thermal behavior, sandwich panels, mineral wool, heat transfer, thermal diffusivity

## Abstract

The paper presents theoretical, experimental and numerical studies on the thermal behavior of mineral wool used in sandwich panels. The aim of this study is to investigate the thermal properties of mineral wool at elevated temperatures and provide a simple model that would allow us to determine the heat propagation in sandwich panels during a fire. The paper proposes a new method to experimentally evaluate thermal diffusivity, derived from theoretical premises. Experiments are conducted in a laboratory furnace where specimens are placed and temperatures inside specimens are measured. Different methods are used to process the test results and calculate the thermal diffusivity of mineral wool. Finally, a numerical analysis of heat transfer using the finite element method (FEM) is performed to validate the obtained thermal properties.

## 1. Introduction

Sandwich panels are commonly utilized in the construction industry. They usually consist of two thin metal sheets (facings) and an insulating core that separates the facings. The core insulation can be either combustible or non-combustible. Sandwich panels shall adhere to various standards and regulations to ensure their safety and reliability. EN 14509 standard [1] specifies requirements for the sandwich panels where the core is made of rigid polyurethane, expanded polystyrene, extruded polystyrene foam, phenolic foam, cellular glass or mineral wool. Additionally, EN 13501-1 standard [2] offers classifications for the fire performance of construction products, including sandwich panels. When there is a need for high classes of fire resistance and a reaction to fire, panels containing mineral wool (MW) core are utilized.

Sandwich panels have both advantages and disadvantages when it comes to fire safety. Their relatively lightweight nature makes them easy to install and transport, which can reduce construction costs and environmental impact. Specific types of insulation materials used in sandwich panels can provide thermal resistance, which may delay the spread of flames and improve overall fire safety. On the other hand, some sandwich panel materials can melt and contribute to the fire’s fuel load, which may intensify the blaze and cause fire to spread more rapidly. Moreover, the insulation materials used in some sandwich panels may release toxic fumes when exposed to heat, posing a significant health hazard to occupants and emergency responders. 

Sandwich panels with an MW core are the subject of this work. Currently, manufacturers deliver their products to a fire laboratory, where they are subjected to tests to determine how long sandwich panels can withstand certain fire conditions in accordance with EN 1364-1 or EN 1365-2 [3,4]. These standards specify procedures for fire resistance tests of construction elements. The whole assembly is tested, which inherently addresses factors, such as fixing, connections between panels, thermal expansion or delamination.

Modelling the fire behavior of sandwich panels allows for using a performance-based design. This, however, requires understanding of the physical phenomena and appropriate definition of material parameters at high temperatures. One of the basic challenges in modelling a sandwich panel exposed to fire is to determine the temperature-dependent thermal properties of a core material. There are three key thermal parameters that are relevant to fire behavior: thermal conductivity (λ), mass density (ρ) and specific heat ($C_{p}$). Nevertheless, the engineering approach to calculating transient heat propagation in a sandwich panel can be based on information about thermal diffusivity. Thermal diffusivity is the quantity that combines all the thermal properties mentioned above (λ, ρ, $C_{p}$). The thermal diffusivity (α) is crucial because it directly affects the heat flow described by the heat transfer differential equation. It is shown below that on the basis of a relatively simple experimental setup, the effective thermal diffusivity of an insulating core material can be assessed, which can then be used to model the heat transfer process.

The aim of the article is to present a simple experimental method that allows us to determine the thermal diffusivity of mineral wool as a function of temperature (θ). Since we are interested in the issues of heat flow in fire load situations, due to large temperature changes, the issue of thermal diffusivity dependence on temperature is very important. Insulating materials, such as mineral wool, are characterized by very low thermal diffusivity, which causes significant problems in determining its value. In the article, we emphasize the simplicity of the method at the expense of some simplifications. The developed method was validated numerically.

## 2. Literature Review

Insulation materials play a significant role in a wide range of applications. The thermal properties of insulation materials are critical for their effectiveness in fire conditions. [5]. The subject of research presented in the article is mineral wool. Over the years, several authors focused on determining the thermal properties of mineral wool or assemblies containing mineral wool and other materials, such as gypsum boards, thin metal sheets or plasterboards [6,7,8]. The thermal conductivity of mineral wool with a density of 80 kg/m^3^ was derived in [9] using a prediction-correction method. This method was applied to fit the computation results to those obtained during standard fire tests. However, the paper does not specify the orientation of the fibers, the number of measurement points or the geometry of the sample. In [10], a detailed thermal analysis of mineral wool in nitrogen and oxygen atmospheres was conducted. The authors reported two exothermic peaks and attributed them to the burning of the binder and crystallization of the amorphous material. The thermal behavior of mineral wool was investigated and modelled in [11,12]. In [11], a multiphysics model of heat and mass transfer coupled with the chemical decomposition of the organic content was developed while in [12] models for predicting the temperature of the unexposed side of sandwich composites made of mineral wool with a stainless steel or plasterboard cladding were presented.

The thermal properties of insulation materials vary depending on their type. At ambient temperature, mineral wool exhibits thermal conductivity in the range of 0.030–0.046 W/(m·K) [13]. In the case of elevated temperature, different values of thermal conductivity of mineral wool are given in various studies. For example, a constant value of 0.036 W/(m·K) was utilized in [9], whereas in [14,15], different thermal conductivity versus temperature functions were used. It is crucial to consider the relationship between thermal conductivity and temperature to accurately evaluate the effectiveness of insulation materials. 

**Figure 1:**
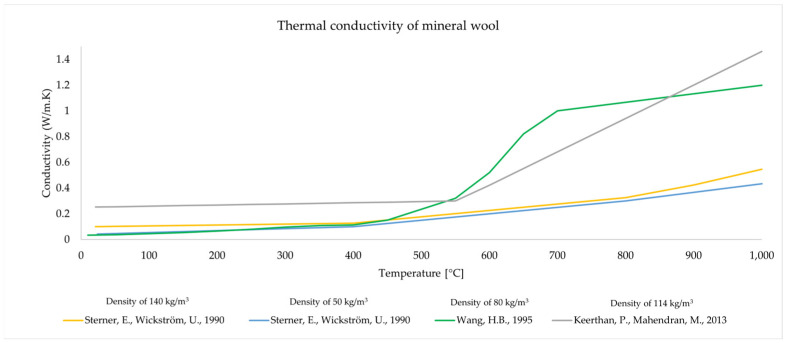
Thermal conductivity of mineral wool as a function of temperature [6,14,15].

There are several sources as [14,15,16] that give MW conductivity as a function of temperature, but they refer to different material densities. There is no standard function that determines the conductivity of MW as a function of temperature (and wool density) or a simple test method that can be used to determine thermal conductivity as a function of temperature. Determining the λ−θ relationship is very difficult because during the production of mineral wool, various types of binders and chemical preparations are used to glue or bind mineral fibers, which affect the thermal processes occurring in the material. To illustrate the problem, the dependence of thermal conductivity on temperature (and density) according to sources [6,14,15] is presented in Figure 1. The graphs show the large differences in thermal conductivity observed in various studies. This can be attributed to differences in the structure of the material or the chemical composition of the materials, but the reason should also be sought in the use of different test methods. In the case of ambient temperature, differences may also result from the humidity of insulating materials [17].

Another material parameter is specific heat. The difficulty of measuring the specific heat of insulating materials results from the fact that these materials are porous and have low thermal conductivity and low heat capacity. In [6,18], a constant value of the specific heat of mineral wool is given, equal to 850 J/(kg·K). The density of mineral wool can also be affected by temperature; insulating materials tend to decrease in density slightly with increasing temperature. This is primarily due to thermal expansion; the material absorbs heat and expands, resulting in lower density. The analysis of available publications shows that, currently, the most general and useful source of mineral wool properties at elevated temperature is the upcoming Eurocode prEN 1995-1-2 (design of timber structures state for 2022) [19], in which the effective thermal properties of mineral wool are generalized based on fundamental experimental and numerical analyses (Table 1). 

Different laboratory methods can be utilized to measure thermal properties, including Transient Plane Source (TPS) [20], Heat Flow Meter Apparatus [5] or Guarded Hot Plate (GHP) [21]. Each of these methods has its advantages, but also notable limitations with regard to the transient properties that are of interest in our study. The heat transfer inside nonporous and nontransparent materials takes place via conduction. For porous materials, heat is transmitted by conduction, radiation and convection. Decoupling those three ways of heat transfer for the assessment of temperature in mineral wool is impractical. Moreover, the measured thermal conductivity will vary with test conditions and test methods (e.g., transient and steady-state methods). According to [16], the applicability of the thermal conductivity obtained with any particular method is limited to heat flow patterns similar to those used in the measurement method. Therefore, typical methods used for insulation materials, such as guarded hot plate and heat flow meter, are applicable when steady-state values are of interest. For some applications, transient methods are used, for example, with the TPS method. During a fire, the conditions are far from a steady-state and differ from typical transient tests. Due to the above-mentioned issues, establishing the so-called effective thermal properties is a prerequisite for building materials. The effective properties are meant to give a good agreement between the experimental data and the calculations [22,23] without the burden of complicated calculations.

None of the methods described above will be used in this study. The proposed method is not expensive and does not require specialized equipment. The method proposed involves the use of a large heat source that is applied to one surface of the specimen, forcing one-dimensional (1-D) heat transfer. The temperature is measured at several points in the sample with thermocouples located in known localizations, and the heat diffusivity is calculated from the time-dependent temperature profile. The simplicity of the method proposed in this study will allow for an analysis of the influence of fiber orientation during the heating process and the influence of chemical reactions inside the specimen. In the next sections, the test method is described, and the validation process with the results is illustrated.

## 3. Description of the Test Method

### 3.1. Heat Diffusion Equation

The heat diffusion equation in the Cartesian coordinate system x, y, z can be presented as:

(1) $$\frac{\partial }{\partial x}\left(\lambda_{x}\frac{\partial \theta }{\partial x}\right)+\frac{\partial }{\partial y}\left(\lambda_{y}\frac{\partial \theta }{\partial y}\right)+\frac{\partial }{\partial z}\left(\lambda_{z}\frac{\partial \theta }{\partial z}\right)+q=\rho C_{p}\frac{\partial \theta }{\partial t},$$

where ρ and $C_{p}$ denote mass density and specific heat (at constant pressure) of the material, respectively. The temperature θ is a function of time t; in general, this function will be different depending on the considered point in space. The term q expresses the rate at which energy is generated per unit volume of the medium (W/m^3^), whereas the right side of Equation (1) expresses the time rate of change of the sensible (thermal) energy of the medium per unit volume. The first three terms of (1) result from the change of the conduction heat rates evaluated using Fourier’s law. Different thermal conductivities $\lambda_{x}$, $\lambda_{y}$, $\lambda_{z}$ are associated with conductions in the *x*-, *y*- and *z*-direction, respectively.

If internal heat sources are eliminated, q can be omitted from Equation (1). If, in addition, the conduction heat rate associated with one direction is much greater than in the other two directions, then Equation (1) reduces to a 1-D problem:

(2) $$\frac{\partial }{\partial x}\left(\lambda_{x}\frac{\partial \theta }{\partial x}\right)=\rho C_{p}\frac{\partial \theta }{\partial t}.$$

In the case of constant thermal conductivity $\lambda_{x}$, which is independent of the position variable, the heat equation is simplified to:

(3) $$\frac{\partial^{2}\theta }{\partial x^{2}}=\frac{1}{\alpha }\frac{\partial \theta }{\partial t},$$

where the thermal diffusivity α is:

(4) $$\alpha =\frac{\;\lambda_{x}}{\rho C_{p}}.$$

In general, it can be assumed that all quantities in Equation (4) depend on temperature. 

The method presented in the article is based on the use of Equation (3) to determine the thermal diffusivity as a function of temperature. Knowledge of such a function makes it possible to model thermal phenomena occurring in variable thermal conditions. In order to apply the proposed method, efforts were made to eliminate internal energy sources and ensure 1-D heat flow. Inaccuracies resulting from simplifications were also estimated.

### 3.2. Experimental Setup

The experimental study involved a cuboid specimen composed of a tested material that was subjected to the heating in the Carbolite Gero CWF 13/36 laboratory-grade chamber furnace (Manufacturer: Carbolite Gero, Hope Valley, UK). This furnace was specifically designed for high-temperature applications, with a maximum operating temperature of 1300 °C and a heating chamber measuring 320 mm × 250 mm × 450 mm, providing a total volume of 36 L. The furnace was equipped with silicon carbide heating elements, known for their superior thermal stability and temperature uniformity. The furnace temperature was controlled by a CC-T1 touch screen temperature controller, offering precise control over 24 segments that could be set as ramp, step or dwell and configured to control relays. The furnace’s safety over-temperature controller provided protection against overheating. 

**Figure 2:**
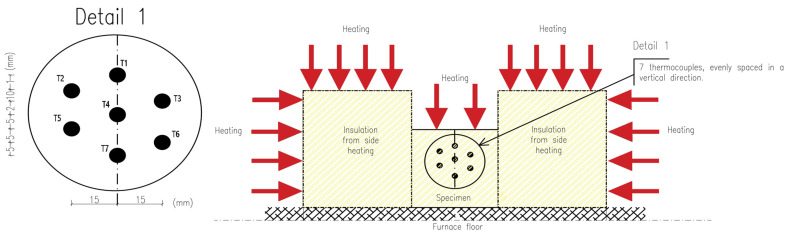
Sketch of a specimen inside the furnace.

**Figure 3:**
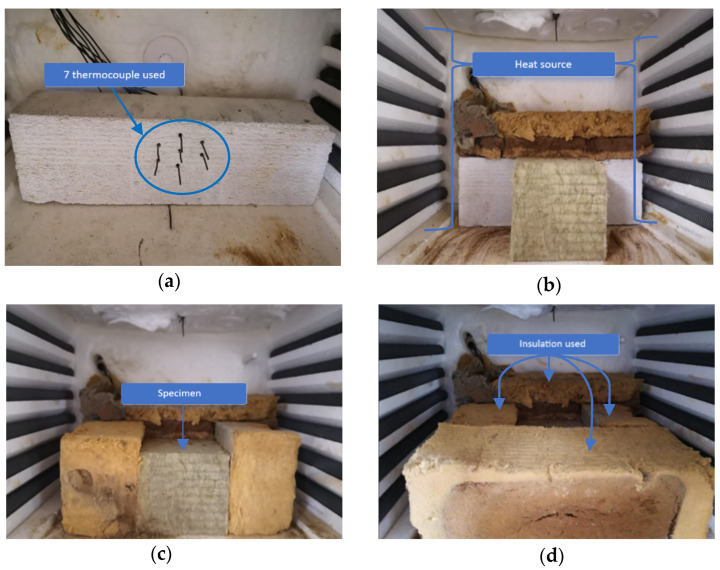
The test specimen inside the electrical furnace: (**a**) fixing position of a thermocouple by the use of an aerated concrete brick at the back of the furnace; (**b**) sticking a mineral wool specimen on the thermocouples; (**c**,**d**) insulating the specimen from all-around.

To minimize lateral heat flow during the heat process, the cuboid specimen being tested was surrounded by insulation material on the front, left and right sides. On the back surface, an aerated concrete brick adhered to the sample, which allowed for precise fixation of thermocouples (see Figure 2 and Figure 3). The sample was exposed from the top, so heat flow was expected from top to bottom. The tests were carried out for samples of 100 mm × 100 mm × 100 mm. Seven type K thermocouples (denoted as T1, T2, T3, T4, T5, T6, T7) were placed inside the specimen at known vertical intervals as shown in Figure 2 (Detail 1) and Figure 3a. PicoLog 6 software (ver. 6.2.6) in conjunction with the TC-08 Pico Data Acquisition (DAQ) devices (Manufacturer: Pico Technology, St Neots, UK) offered a comprehensive solution for capturing, recording, analysing and visualising data from thermocouples placed inside the tested specimens. This allowed for effortless monitoring and recording of temperature data from all thermocouples at 1-s intervals. Once the data were captured, PicoLog 6 software enabled the secure storage of the acquired temperature measurements. This ensured that valuable data was preserved for analysis.

Due to the small dimensions of the sample, the thermocouples were horizontally shifted relative to each other. However, it is worth noting that for 1-D heat transfer, isotherms in the specimen were horizontal; hence, only the vertical coordinate of the measurement point was relevant. The horizontal offset of the thermocouples allowed for their vertical distances to be smaller. The true locations of thermocouples were precisely verified after each test to provide the reliable data for the calculation. 

### 3.3. Calculation Method

This section aims to present the methodology employed for calculating the diffusivity and provide the calculation process for a specific time point. Using Equation (3), the function of the thermal diffusivity α(θ) can be obtained knowing the 2nd order derivative of the temperature with respect to distance coordinate and the derivative of the temperature with respect to time:

(5) $$\alpha \left(\theta \right)=\left(\frac{\partial \theta }{\partial t}\right)\cdot {\left(\frac{\partial^{2}\theta }{\partial x^{2}}\right)}^{-1}.$$

Experimental data provided spatial and temporal distribution of temperature in a material at distinct positions and specified time steps. It allowed for the approximation of the derivatives in Equation (5) based on the experimental results and then the calculation of the thermal diffusivity.

To calculate the thermal diffusivity, two approaches were investigated to determine the approximate derivatives in Equation (5). The first approach was to apply finite differences with high order of precision. In the second approach, differentiable regression curves for the spatial and temporal progression of temperature were used. The finite differences approach, being the most straight-forward, collapsed for calculation of $\partial^{2}\theta /\partial x^{2}$. It was due to limited number of temperature points and high thermal gradient in the direction of heat flow. The experimental data showed some noise, which was amplified when calculating the derivatives. This was avoided in the second approach. The use of regression curves made the temperature function and its derivatives continuous and smooth. Hence, the second approach was used and illustrated hereafter.

**Figure 4:**
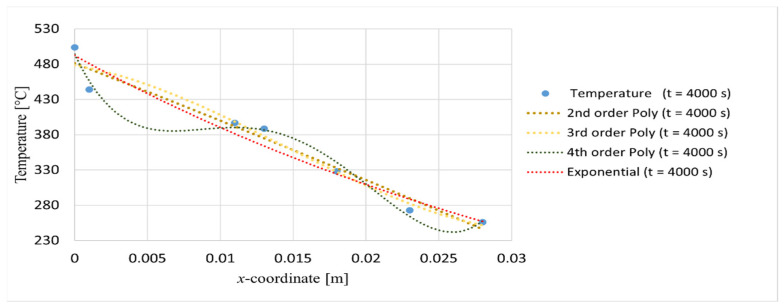
Regression curves for time *t* = 4000 s.

At each time step, at selected points in the specimen, the temperature was measured using thermocouples. Seven thermocouples labelled T1–T7 were used in the following tests. To calculate, four types of regression curves were initially selected: 2nd order polynomial, 3rd order polynomial, 4th order polynomial and exponential function. Based on the equation of each regression curve, the second derivative was determined. Exemplary results for the time *t* = 4000 s are shown in Figure 4 and Table 2. Although the results presented in Figure 4 and Table 2 were derived from a single time step of a specific experiment (Test 2) and at specific node (T2), they were intentionally chosen as representative examples. The issues of instability or temperature fluctuations observed in these data are common but should be considered in a broader context.

The equations of the regression curves presented in Figure 4 are given in Table 2. The values of the second derivative vary significantly depending on the type of regression curve used. The use of polynomials for regression, as shown in Figure 4, can result in an inflection point in the analyzed range. As long as the specimen heats up on one side, the existence of an inflection point is physically incorrect. Thus, the inflection points seen in Figure 4 were related to experimental measurement noise. The noise was associated with high-frequency measurements using thermocouples. With this in mind, the exponential function was chosen as the one that gave the most reliable temperature profile inside the specimen. In exponential regression, the value of the second derivative was always of the same sign (no inflection points of the regression curve). In addition, Equation (3) is a hyperbolic partial differential equation that can be solved analytically in some simplified cases, and the analytical solution is often in the form of an exponential function [24,25].

**Figure 5:**
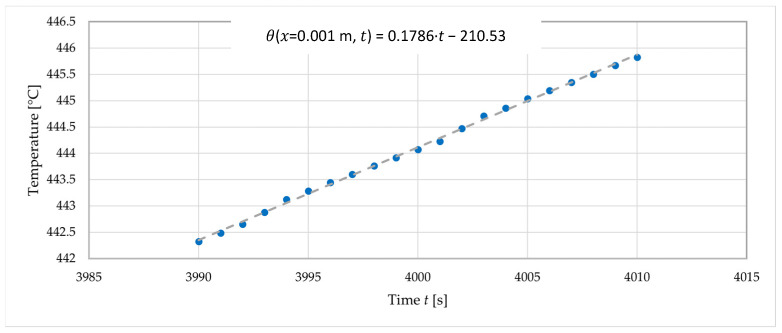
An example of the time–temperature relationship and the corresponding linear regression for a given measurement point T2 (*x* = 0.001 m).

Regression was also used to provide the derivative of temperature over time. Figure 5 shows the graph of the time–temperature relationship for an example measurement point and the linear regression for this data. The regression corresponding to the time *t* = 4000 s is built for a 20 s time interval from *t* = 3990 s to *t* = 4010 s. In a similar way, for the time *t* = 4001 s, it will be the interval from *t* = 3991 s to *t* = 4011 s. Using linear regression, for time *t* = 4000 s one obtains:

(6) θ(x=0.001 m,t )=0.1786·t−210.53,

(7) ∂θ(x=0.001 m,t )/∂t=0.1786.

From the determined value of the derivative of temperature over time and the exponential regression results from Table 2, the thermal diffusivity was determined for the measurement points (T2–T6) and for the specific time *t* = 4000 s, which corresponded to a specific temperature θ. The results are presented in Table 3.

The same methodology used to calculate the thermal diffusivity at node T2 was applied to the other nodes T3, T4, T5 and T6. The results are presented in Table 3 for thermal diffusivity for all node T2–T6 and for the specific time *t* = 4000 s. The determined values of the thermal diffusivity corresponded to the specified temperatures θ. 

### 3.4. Reduction of 3-D Problem to 1-D Problem

The assumption of 1-D heat transfer in the analysed region was verified numerically. To ensure that the diffusion of the heat is predominantly within the *x*-direction, it was necessary to analyse the derivatives of the heat flux in all directions in coordinates related to real positions of thermocouples and confirm that the derivative of the heat flux in the *x*-direction was significantly greater than the derivatives of the heat fluxes in the *y*- and *z*-directions.

**Figure 6:**
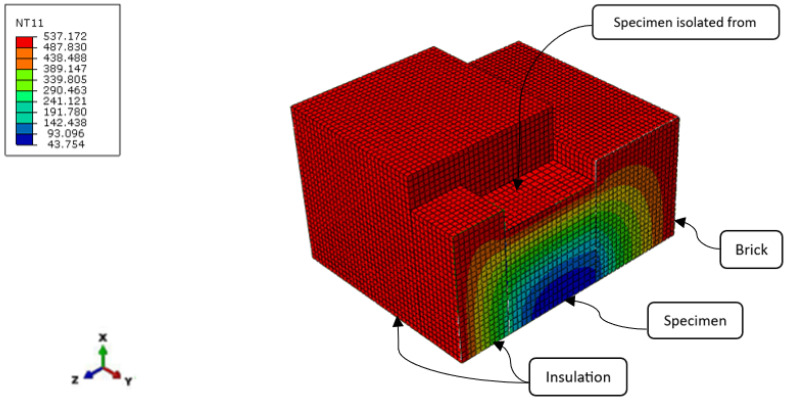
Cross sectional view–nodal temperatures for *t* = 53 min at the temperature of heated surfaces 537 °C.

The 3-D model of the heat transfer according to the test method described above was developed in ABAQUS software. The cross-sectional view of the model is given in Figure 6. To analyse the heat transfer problem, DC3D8 (8-node linear brick) finite elements were applied. The material property, i.e., the thermal diffusivity, which depended on temperature, was based exactly on the results of this paper. Chronologically, this analysis was done at the end; however, for the clearness of this paper, it is presented here. In the numerical model, the tie constraint was used between the specimen surfaces and the surfaces of the insulation and brick. For this analysis, the boundary conditions were defined as heat fluxes due to convection and radiation on all surfaces. The heat fluxes’ boundary condition was applied at the surfaces that did not have contact with the specimen, such as the external surfaces of brick element, the external surfaces of insulation and also the top surface of the specimen. The mesh sensitivity analysis revealed that the mesh size of 0.005 m was sufficient for the analysis. The temperature defined on the heated surfaces was equal to the one captured inside the furnace during the test. The initial temperature was predefined as 21 °C.

**Figure 7:**
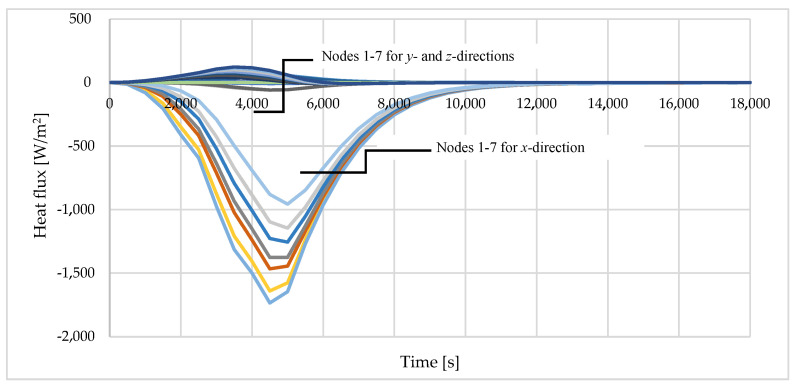
Numerical result of the heat flux on *x-*, *y*- and *z*-directions of the specimen tested.

Figure 7 presents the magnitude of the heat flux components (*x*, *y* and *z*) for points T1–T7. The results were obtained from numerical analysis. It is clear from the illustration below that the amount of the heat transfer in the *x*-direction was much greater than the heat flux in the *y*- and *z*-direction. However, the comparison of the expressions $\frac{\partial }{\partial x}\left(\lambda_{x}\frac{\partial \theta }{\partial x}\right)$, $\frac{\partial }{\partial y}\left(\lambda_{y}\frac{\partial \theta }{\partial y}\right)$ and $\frac{\partial }{\partial z}\left(\lambda_{z}\frac{\partial \theta }{\partial z}\right)$ was needed to show that the 1-D heat flow assumption was close to reality.

To compare the partial derivatives $\frac{\partial }{\partial x}\left(\lambda_{x}\frac{\partial \theta }{\partial x}\right)$, $\frac{\partial }{\partial y}\left(\lambda_{y}\frac{\partial \theta }{\partial y}\right)$ and $\frac{\partial }{\partial z}\left(\lambda_{z}\frac{\partial \theta }{\partial z}\right)$, the numerical model was applied, in which nodes were evenly spaced in each spatial direction (distances between nodes were: Δx=0.02 m, Δy=0.02 m and Δz=0.02 m). The terms in the brackets represent the components of heat flux (e.g., $\lambda_{x}\frac{\partial \theta_{i}}{\partial x}=-q_{x_{i}}$). To determine the increments of these components in node *i*, the heat flux values q in the neighboring nodes and the finite difference method were used. For the *x*-direction, it is:

(8) $$\frac{\partial }{\partial x}\left(\lambda_{x}\frac{\partial \theta_{i}}{\partial x}\right)=\frac{-q_{x_{i+1}}+q_{x_{i-1}}\;}{2\Delta x},$$

where $q_{x_{i+1}}$ and $q_{x_{i-1}}$ are the heat fluxes in the *x*-direction for the nodes *i* + 1 and *i* − 1, respectively.

**Figure 8:**
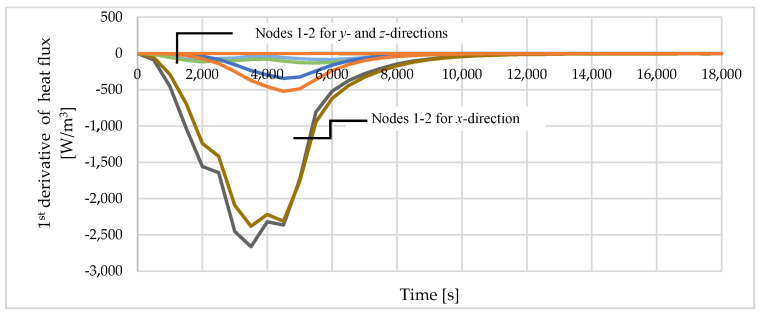
The comparison of the 1st derivatives of the heat flux at nodes T1 and T2 obtained from numerical analysis.

Figure 8 presents the first derivatives of the heat flux according to the *x*-, *y*- and *z*-directions for positions T1 and T2. It is clear that the derivative of the heat flux in the *x*-direction is one order of magnitude greater than in the other directions. The minor deviation in heat flux observed in the *z*-direction at t = 5500 s was attributed to the adhesion of an aerated concrete brick to the tested specimen, which possessed higher thermal conductivity than mineral wool. As a result, it is suggested that the simplified test exhibited certain limitations that needed to be managed in order to derive meaningful conclusions. In future research, this shortcoming can be easily removed by inserting a layer of mineral wool between the brick and the specimen. Based on the presented analysis, it can be concluded that the assumption of a 1-D heat flow was close to reality.

## 4. Experiments

The experimental setup and calculation method described in section above was used. The tested specimens were made of the mineral wool with density of 114 kg/$m^{3}$. The specimens were extracted from a sandwich panel. Two consecutive tests on three samples were performed: Sample A, Sample B and Sample C. The first two samples were assessed with the fibres perpendicular to the furnace floor, as they were aligned in the panels. The third sample (Sample C) was evaluated with fiber orientation parallel to the furnace floor. 

In preliminary tests, it was found that during the heating of the fresh mineral wool, the temperature increased significantly after reaching about 200 °C. This was due to the burning out of the binder that was used in the production of the mineral wool. Therefore, tests on samples A, B and C consisted of two heating cycles. The first, which aimed to capture the effect of binder burning. The second to check the behavior of mineral wool without the burnt binder. A detailed description of the tests is presented in Table 4. The mineral wool used to insulate the tested specimens was pre-heated in order to remove the organic content and prevent it from generating heat that would influence the temperature field within the tested samples. 

After each experimental test, the furnace and samples were cooled about 12 h. In all tests, the temperature was measured at points T1, T2, T3, T4, T5, T6 and T7, for the test period 5 h = 18,000 s, with the values recorded every 1 s. 

## 5. Results

### 5.1. Experimental Results

**Figure 9:**
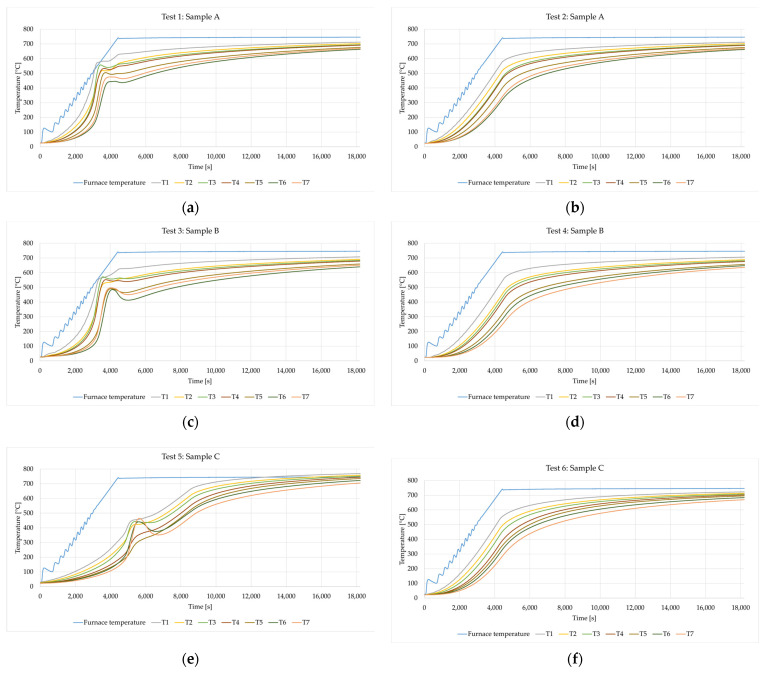
Time–temperature dependence for the tested samples: (**a**) Sample A—first heating cycle; (**b**) Sample A—second heating cycle; (**c**) Sample B—first heating cycle; (**d**) Sample B—second heating cycle; (**e**) Sample C—first heating cycle and (**f**) Sample C—second heating cycle.

The experiments carried out consisted of recording the temperature at the measurement points (T1–T7), where the temperature was a function of time. Figure 9 shows the recorded temperature graphs for samples A, B and C. Figure 9a,c,e correspond to Test 1, Test 3 and Test 5, in which fresh samples were tested. 

In these graphs, the disturbance appears between *t* = 3500 s and *t* = 5500 s. The disturbances are the result of the combustion reaction of the mineral wool binder, which causes an increase in the temperature inside the samples. In the second heating cycle of the samples (Test 2, 4 and 6), such a phenomenon was not noticed. It can therefore be concluded that the process of the sample preheating largely eliminates the heat generation inside the sample during Tests 2, 4 and 6. Once 4400 s have passed, the heating phase comes to an end, and the furnace temperature remains constant. The temperatures within the samples gradually settle into a stable condition (steady state). 

All tests were performed for the same rate of temperature increase in the furnace. Nevertheless, it turned out that the phenomenon of binder burning occurred definitely the latest (after about 1 h and 30 min) in sample C (Test 5), i.e., a sample in which the fibers are parallel to the plane affected by the temperature. In samples A and B, the fibers are perpendicular to the heated plane, which results in faster heat flow and earlier combustion of the mineral wool binder (after about 1 h from the start of the test). Figure 9 conclusively demonstrates that the orientation of fibers significantly impacts the heat transfer characteristics within mineral wool. Hence, the arrangement of fibers also affects the pathways for heat conduction, convection and radiation, influencing how heat is distributed and dissipated through within the material.

### 5.2. Thermal Diffusivity

**Figure 10:**
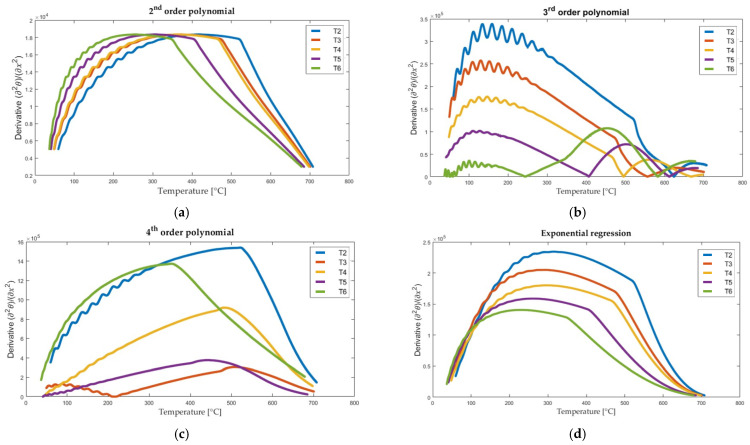
The second derivative of temperature with respect to the spatial coordinate, obtained for Test 2, sample A using the regression curve function: (**a**) 2nd order polynomial; (**b**) 3rd order polynomial; (**c**) 4th order polynomial and (**d**) exponential function.

As it was mentioned in Section 3.3, to determine the thermal diffusivity, it is necessary to calculate the second derivative of the temperature with respect to the spatial coordinate. The possibility of using a second-order polynomial, a third-order polynomial, a fourth-order polynomial and an exponential function was explored. The respective results for Sample A, Test 2, for measurement points T2–T6 are presented in Figure 10. The curves clearly illustrate significant changes in the results of the regression curve. In the case of polynomials of the second and third degree, a large variability of the second derivative is obtained. To obtain a good prediction of thermal diffusivity, the exponential regression was chosen for further investigation. Calculations of the derivative for each recorded time *t*, for points T2–T6, were performed using the MATLAB R2017a software.

A linear regression function determined for a time interval of 20 s was used to determine the temperature derivative with respect to the time variable (see Section 3.3). MATLAB software was used to calculate these derivatives for each recorded time *t_i_*, for points T2–T6.

**Figure 11:**
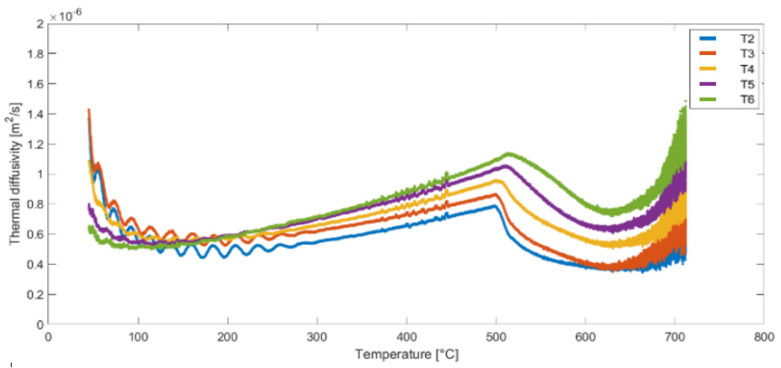
Thermal diffusivity as a function of temperature for nodes T2, T3, T4, T5 and T6—Sample A, Test 2.

**Figure 12:**
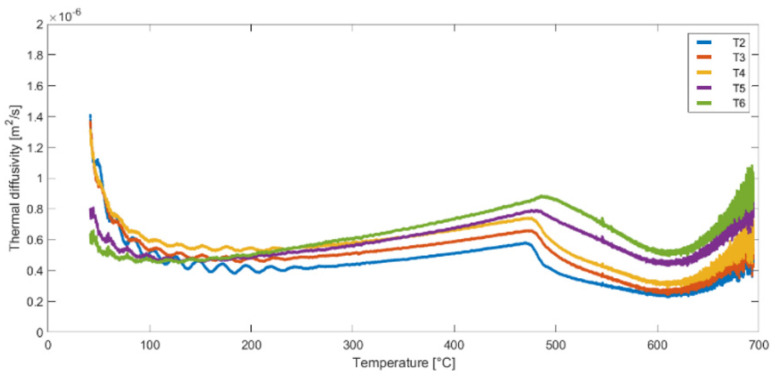
Thermal diffusivity as a function of temperature for nodes T2, T3, T4, T5 and T6—Sample B, Test 4.

**Figure 13:**
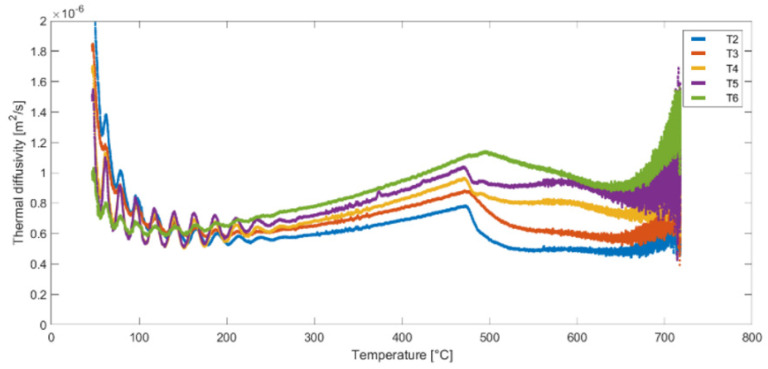
Thermal diffusivity as a function of temperature for nodes T2, T3, T4, T5 and T6—Sample C, Test 6.

Following the completion of the derivative calculations, the thermal diffusivity values for points T2–T6 were determined using Equation (5). The thermal diffusivity results for the second heat cycle of samples (A, B and C) are presented in Figure 11, Figure 12 and Figure 13. These figures illustrate the variation of thermal diffusivity as a function of temperature, providing valuable insights into the heat transfer properties of the materials at different temperature levels. Some differences can be noticed in the results obtained for the same temperature but for different measurement points (T2 to T6).

**Figure 14:**
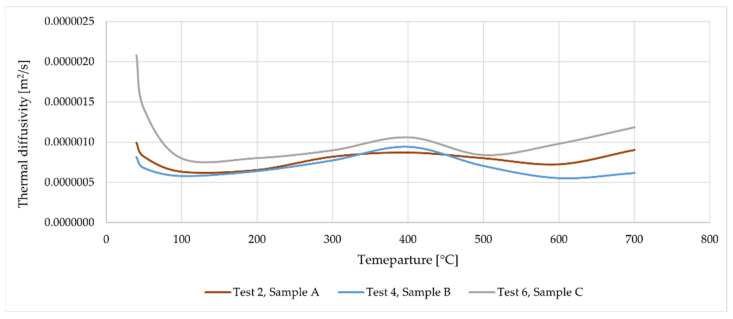
Thermal diffusivity (average value) as a function of temperature.

Figure 14 shows the average value of thermal diffusivity from the curves obtained for measurement points T2–T6. One curve corresponds to one test (Test 2—data from Figure 11, Test 4—data from Figure 12 and Test 6—data from Figure 13), each of these tests was performed on pre-heated samples. This is very important to eliminate internal heat sources before performing the test; calculations performed on data for samples without pre-heating lead to different results. The averaged functions shown in Figure 14 represent the general characteristics of the behavior of mineral wool subjected to temperature treatment. The results obtained for samples A and B are very similar (especially in the range up to 450 °C), which means that the results are characterized by a certain reproducibility. The thermal diffusivity obtained for sample C is higher in the entire temperature range than for the other samples, which once again confirms the influence of fiber orientation on the thermal properties of mineral wool.

## 6. Validation Procedure

**Figure 15:**
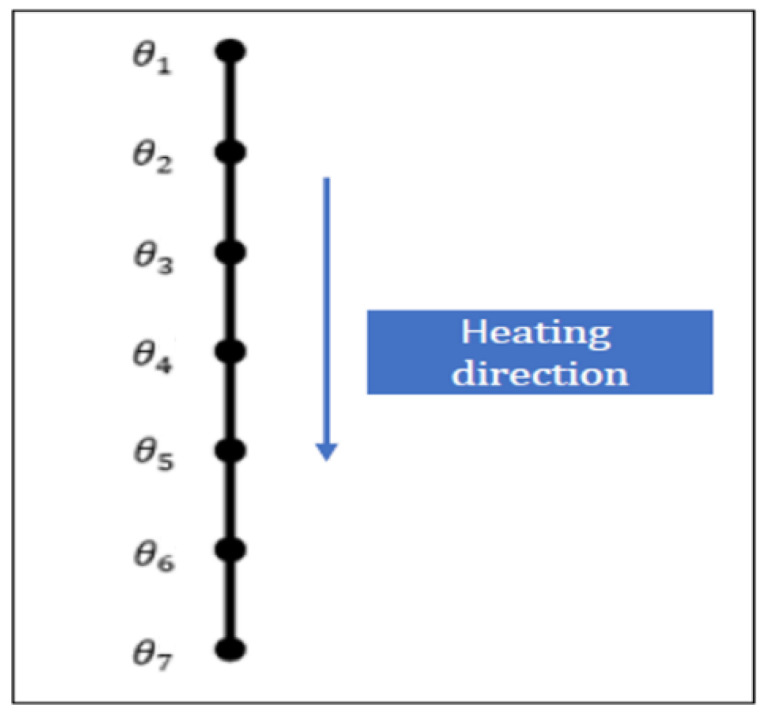
1-D model of heat transfer.

In order to validate the method of determining thermal diffusivity, it is planned to compare the performed experiments with the appropriate numerical model, in which the experimentally determined values of thermal diffusivity will be used. The comparison will refer to the temperature versus time values determined for different points located in the sample, see Figure 15. 

As previously stated, this study utilized a one-dimensional model. The comparison between the numerical analysis and experimental data was conducted to validate the thermal properties of the mineral wool with respect to temperature. To simplify our study’s objective, a planar wire was designed using ABAQUS 2019 software to develop and validate 1-D heat transfer based on the test results. DC1D2 elements were used to model the heat transfer through the mineral wool. The thermal properties of mineral wool were defined by the thermal diffusivity obtained from a specific test (cf. Figure 14). The boundary conditions applied in this numerical simulation are the temperatures at the first node (T1) and the last node (T7), which were obtained in the relevant experiment (Test 2, 4 or 6).

To present the validation of the method described above, Test 4 sample B is used. Test 4 involves the second heating cycle of sample B. During this heating cycle, no temperature disturbances were detected inside the sample, which means that no combustion reaction of the wool fiber binder occurs in the sample.

**Figure 16:**
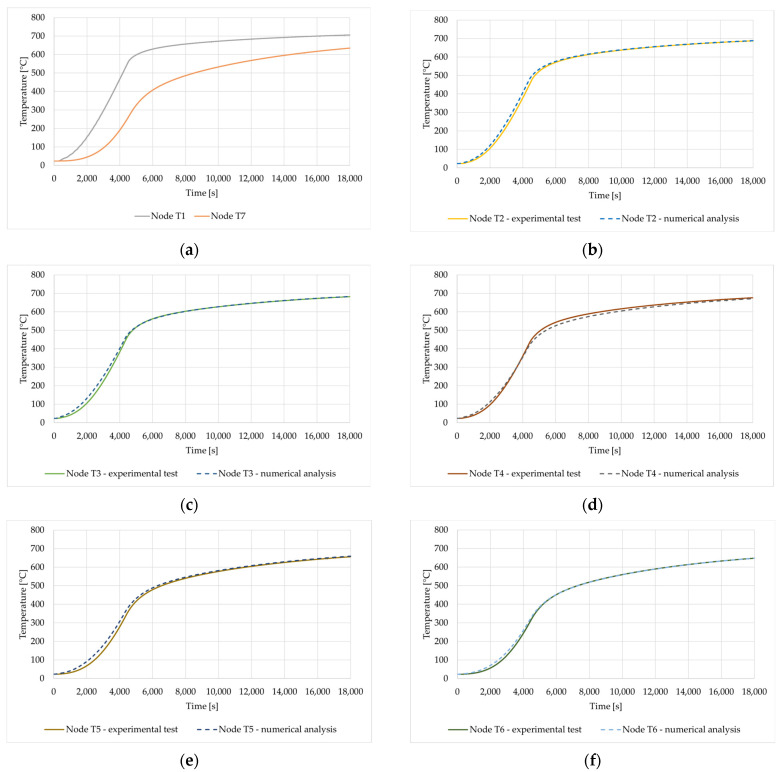
Comparison of the experimental and numerical results: (**a**) Boundary conditions for the numerical problem—temperatures at nodes T1 and T7; (**b**) Time–temperature relationship at node T2; (**c**) Time–temperature relationship at node T3; (**d**) Time–temperature relationship at node T4; (**e**) Time-temperature relationship at node T5 and (**f**) Time–temperature relationship at node T6.

In Figure 16a, the boundary conditions (temperature at points T1 and T7) employed for the numerical study are illustrated. These BCs define the thermal conditions at the boundaries of the specimen during the numerical simulation. Figure 16b–f provide a detailed comparison between the experimentally measured temperature and the corresponding temperature values determined numerically for various points (T2–T6) within the mineral wool. It is clear that the results of the numerical analysis are quite consistent with the experimental tests.

A similar agreement of the experimental and numerical results was obtained for the remaining samples, i.e., for Test 2 (Sample A) and Test 6 (Sample C). The validation process is crucial for verifying the effectiveness of the numerical approach in predicting the temperature behavior of mineral wool under specific boundary conditions. Consistency between experimental and numerical results indicates a successful model that can then be used to predict the thermal behavior of mineral wool.

## 7. Conclusions

This study focused on predicting the thermal behavior of mineral wool used in sandwich panels when exposed to elevated temperatures. For this purpose, a new research method based on temperature measurements in a sample subjected to thermal load was proposed. The results obtained for different points and dependent on time allowed for the experimental determination of the thermal diffusivity as a function of temperature. 

The presented approach required the determination of the second derivative of temperature after the spatial variable. This was accomplished by using a regression curve in the form of an exponential function. It was also required to determine the time derivative of temperature, which was achieved using the linear regression function.

The conducted tests showed that mineral wool has a binder, which is an additional source of heat when exposed to temperature. During laboratory tests, this effect can be eliminated by pre-heating the samples to a temperature of about 200 °C.

Carrying out the planned tests required forcing a relatively simple heat flow. The use of thermal insulation around the sample forced a flow close to one-directional, which was demonstrated and supported by calculations.

Because the samples of the same mineral wool were tested but in a different fibre orientation, different results of the thermal diffusivity function were obtained. Thus, it was shown that the orientation of the fibers affects thermal parameters (mineral wool should be treated as an anisotropic material), but the presented method is suitable for determining the thermal diffusivity function regardless of the direction of heat flow. The presented experimental method has been validated numerically. The method does not require complicated equipment and is easy to use and interpret. It should be treated as an alternative solution to the existing methods of determining thermal parameters of insulating materials.

The results of the presented experiments emphasize the sensitivity and importance of thermal properties, which must be considered as a function of temperature when a material is exposed to high temperatures. This is of great importance for the correct modelling of phenomena occurring during a fire.

## Figures and Tables

**Table 1:** Thermal properties of mineral wool insulation with density more than 26 kg/m^3^ [19].

Temperature [°C]	*λ* [W/m·K]	*C_p_* [kJ/(kg·K]	$\rho /\rho_{20}$ [-]
20	0.036	0.880	1.00
100	0.047	1.040	1.00
200	*	1.160	0.980
400	$0.09\;\cdot \left(11\;\cdot \;e^{-0.05\;\cdot \;\rho_{20}}+1.9\right)$	1.280	0.977
600	$0.15\;\cdot \left(11\;\cdot \;e^{-0.05\;\cdot \;\rho_{20}}+1.9\right)$	1.355	0.973
800	$0.23\;\cdot \left(11\;\cdot \;e^{-0.05\;\cdot \;\rho_{20}}+1.9\right)$	1.430	0.970
925	$0.30\;\cdot \left(11\;\cdot \;e^{-0.05\;\cdot \;\rho_{20}}+1.9\right)$	1.477	0.967
1200	$0.45\;\cdot \left(11\;\cdot \;e^{-0.05\;\cdot \;\rho_{20}}+1.9\right)$	1.580	0.88

* Linear interpolation can be applied.

**Table 2:** Exemplary calculation of derivatives at selected time step and selected point.

2nd order polynomial regression	$\theta \left(x,\;t=4000\;s\right)=-14,924\;x^{2}-7982.7\;x+481.37\;\theta^{\prime \prime }\left(x,\;t=4000\;s\right)=-14,924$	
	Node T2	$\theta^{\prime \prime }\left(x=0.001\;m,\;t=4000\;s\right)=-14.924$
	Node T3	$\theta^{\prime \prime }\left(x=0.011\;m,\;t=4000\;s\right)=-14.924$
	Node T4	$\theta^{\prime \prime }\left(x=0.013\;m,\;t=4000\;s\right)=-14.924$
	Node T5	$\theta^{\prime \prime }\left(x=0.018\;m,\;t=4000\;s\right)=-14.924$
	Node T6	$\theta^{\prime \prime }\left(x=0.023\;m,\;t=4000\;s\right)=-14.924$
3rd order polynomial regression	$\theta \left(x,\;t=4000\;s\right)=\left(1\times 10^{7}\right)\;x^{3}-485,786\;x^{2}-3263.7\;x+478.1\;\;\theta^{\prime \prime }\left(x,\;t=4000\;s\right)=60,000,000\;x-971,572$	
	Node T2	$\theta^{\prime \prime }\left(x=0.001\;m,\;t=4000\;s\right)=-911,572$
	Node T3	$\theta^{\prime \prime }\left(x=0.011\;m,\;t=4000\;s\right)=-311,572$
	Node T4	$\theta^{\prime \prime }\left(x=0.013\;m,\;t=4000\;s\right)=191,572$
	Node T5	$\theta^{\prime \prime }\left(x=0.018\;m,\;t=4000\;s\right)=108,428$
	Node T6	$\theta^{\prime \prime }\left(x=0.023\;m,\;t=4000\;s\right)=408,428$
4th order polynomial regression	$\theta \left(x\;,\;t=4000\;s\right)=\left(6\times 10^{9}\right)\;x^{4}-\left(3\times 10^{8}\right)\;x^{3}+\left(6\times 10^{6}\right)\;x^{2}-43,843\;x+494.32\;\theta^{\prime \prime }\left(x\;,\;t=4000\;s\right)=72,000,000,000\;x^{2}-1,800,000,000\;x+12,000,000$	
	Node T2	$\theta^{\prime \prime }\left(x=0.001\;m,\;t=4000\;s\right)=-911,572$
	Node T3	$\theta^{\prime \prime }\left(x=0.011\;m,\;t=4000\;s\right)=-311,572$
	Node T4	$\theta^{\prime \prime }\left(x=0.013\;m,\;t=4000\;s\right)=-191,572$
	Node T5	$\theta^{\prime \prime }\left(x=0.018\;m,\;t=4000\;s\right)=108,428$
	Node T6	$\theta^{\prime \prime }\left(x=0.023\;m,\;t=4000\;s\right)=408,428$
Exponential regression	$\theta \left(x\;,\;t=4000\;s\right)=492.25\;e^{-23.2\;x}\;\;\theta^{\prime \prime }\left(x\;,\;t=4000\;s\right)=264,948.64\;e^{-23.2\;x}$	
	Node T2	$\theta^{\prime \prime }\left(x=0.001\;m,\;t=4000\;s\right)=258,872.58$
	Node T3	$\theta^{\prime \prime }\left(x=0.011\;m,\;t=4000\;s\right)=205,272.01$
	Node T4	$\theta^{\prime \prime }\left(x=0.013\;m,\;t=4000\;s\right)=195,964.98$
	Node T5	$\theta^{\prime \prime }\left(x=0.018\;m,\;t=4000\;s\right)=174,501.96$
	Node T6	$\theta^{\prime \prime }\left(x=0.023\;m,\;t=4000\;s\right)=155,389.67$

**Table 3:** Thermal diffusivity calculation for *t* = 4000 s and given measurement points (T2–T6).

Thermal Diffusivity for *t* = 4000 s	$\alpha \left(\theta \right)=\frac{\partial \theta }{\partial t}/\frac{\partial^{2}\theta }{\partial x^{2}}$	
	Node T2	$\alpha \left(x=0.001\;m,\;t=4000\;s\right)=0.1786\;:258,872.58=6.90\times 10^{-7}$(m^2^/s)
	Node T3	$\alpha \left(x=0.011\;m,\;t=4000\;s\right)=0.1765\;:205,272.01=8.60\times 10^{-7}\;$(m^2^/s)
	Node T4	$\alpha \left(x=0.013\;m,\;t=4000\;s\right)=0.1750\;:195,964.98=8.93\times 10^{-7}$(m^2^/s)
	Node T5	$\alpha \left(x=0.018\;m,\;t=4000\;s\right)=0.1644\;:174,501.96=9.42\times 10^{-7}$(m^2^/s)
	Node T6	$\alpha \left(x=0.023\;m,\;t=4000\;s\right)=0.1521\;:155,389.67=9.79\times 10^{-7}$(m^2^/s)

**Table 4:** Description of performed tests.

Experiments	Heat Rate	Durations	
Test 1, Sample A (density of 114 kg/m^3^)	10 °C per 60 s	5 h	Test 1: fresh sample Heating from 21 °C to 750 °C during a period of 4400 s, then a constant temperature of 750 °C for the rest of duration.
Colling Sample A	Room Temperature	12 h	Cooling to room temperature.
Test 2, Sample A (density of 114 kg/m^3^)	10 °C per 60 s	5 h	Test 2: sample after Test 1 Heating from 21 °C to 750 °C during a period of 4400 s, then a constant temperature of 750 °C for the rest of duration.
Test 3. Sample B (density of 114 kg/m^3^)	10 °C per 60 s	5 h	Test 3: fresh sample Heating from 21 °C to 750 °C during a period of 4400 s, then a constant temperature of 750 °C for the rest of duration.
Colling Sample B	Room Temperature	12 h	Cooling to room temperature.
Test 4, Sample B (density of 114 kg/m^3^)	10 °C per 60 s	5 h	Test 4: sample after Test 1 Heating from 21 °C to 750 °C during a period of 4400 s, then a constant temperature of 750 °C for the rest of duration.
Test 5, Sample C (density of 114 kg/m^3^)	10 °C per 60 s	5 h	Test 5: fresh sample Heating from 21 °C to 750 °C during a period of 4400 s, then a constant temperature of 750 °C for the rest of duration.
Colling Sample C	Room Temperature	12 h	Cooling to room temperature.
Test 6, Sample C (density of 114 kg/m^3^)	10 °C per 60 s	5 h	Test 6: sample after Test 1 Heating from 21 °C to 750 °C during a period of 4400 s, then a constant temperature of 750 °C for the rest of duration.

## Data Availability

The data presented in this study are available on request from the corresponding author.
